# Urinary System Manifestation of IgG4-Related Disease: Clinical, Laboratory, Radiological, and Pathological Spectra of a Chinese Single-Centre Study

**DOI:** 10.1155/2020/5851842

**Published:** 2020-07-03

**Authors:** Fei Teng, Hui Lu, Ke Zheng, Gang Chen, Yubing Wen, Zheng Liu, Linyi Peng, Li Huo, Xiaofeng Zeng, Wen Zhang, Xuemei Li

**Affiliations:** ^1^Department of Internal Medicine, Peking Union Medical College Hospital, Chinese Academy of Medical Science & Peking Union Medical College, Beijing, China; ^2^Department of Rheumatology, Peking Union Medical College Hospital, Chinese Academy of Medical Science & Peking Union Medical College, National Clinical Research Center for Dermatologic and Immunologic Diseases (NCRC-DID), Beijing, China; ^3^Department of Nephrology, Peking Union Medical College Hospital, Chinese Academy of Medical Science & Peking Union Medical College, Beijing, China; ^4^Department of Nuclear Medicine, Peking Union Medical College Hospital, Chinese Academy of Medical Science & Peking Union Medical College, Beijing, China

## Abstract

**Background:**

IgG4-related disease is a new disease entity, but little attention was drawn to urinary system involvement besides nephritis or nephropathy. Here, we described clinical, radiological, and pathological manifestations of IgG4-related urinary disease (IgG4-RUD) and assess its treatment responses.

**Methods:**

We conducted a retrospective study enrolling 65 IgG4-RUD patients from an IgG4-related disease (IgG4-RD) cohort of the Peking Union Medical College Hospital. Clinical, laboratory, radiological, pathological data were collected, and treatment response to immunosuppressants were analysed.

**Results:**

IgG4-related interstitial nephritis (TIN, 32.3%), glomerular nephritis (GN, 7.7%), renal pelvis and ureter involvement (21.5%), abnormal radiology with quiescent clinical presentation (13.8%), and renal parenchymal lesion plus retroperitoneal fibrosis (RPF, 18.5%) were major lesion types of IgG4-RUD. All patients had elevated serum IgG4, 76.9% had hyperglobulinemia, and 92.3% had elevated serum IgE at diagnosis. IgG4-TIN patients presented with renal dysfunction, and 94.3% had low serum complement C3 and IgG4-GN presented with nephrotic syndrome, while renal pelvis and ureter involvement had normal renal function and urinalysis. IgG4-RPF with renal parenchymal involvement presented with acute renal dysfunction and required emergency medical intervention. Renal cortex low-density areas, parenchyma or pelvis nodular mass, bilateral enlargement of the kidney, and renal pelvis and ureter mass/wall thickening were specific image patterns of IgG4-RUD. Infiltration of plasma lymphocytes and storiform fibrosis were histopathological features of IgG4-RUD. Patients showed satisfactory responses to immunosuppressive treatment, but complete recovery of renal function was difficult to achieve in IgG4-TIN. Four patients (6.2%) experienced clinical relapses during the maintenance period.

**Conclusion:**

IgG4-RUD had diverse lesion types and distinctive manifestations. Radiological examinations were helpful for diagnosis and treatment evaluation. Patients showed good initial response to immunosuppressive treatment but relapses could occur at the maintenance period.

## 1. Introduction

IgG4-related disease (IgG4-RD) is an increasingly known systemic clinicopathological entity that is characterized by elevated serum IgG4 levels, tumescent enlargement of multiple organs, diffuse infiltration of plasma lymphocytes, and storiform fibrosis in lesioned organs. IgG4-RD was first proposed by Kamisawa et al. in 2003 [1] and was formally acknowledged by academia later in 2010 [2], with many previously independent diseases such as Mikulicz disease, Kuttner tumour, and Riedel thyroiditis being recognized as disease entities [3]. While pancreas and exocrine gland involvement in IgG4-RD is under the spotlight, few systematic studies focus on urinary system involvement, which accounts for an important proportion of IgG4-RD [4–8].

The first case of IgG4-related kidney disease was tubulointerstitial nephritis (TIN), reported as an accompanying condition with autoimmune pancreatitis (AIP) in 2004 [9]. Since then, emerging reports of membranous nephropathy (MN) and other forms of glomerulonephritis (such as membranoproliferative nephritis, IgA nephropathy, and Henoch-Schonlein purpura nephritis) have diversified the forms of kidney lesions that were later recognized as IgG4-related kidney disease (IgG4-RKD) [10–12]. In addition to IgG4-RKD, the renal pelvis, ureter, and bladder were also reported as lesion locations in the urinary system of IgG4-RD [13–17]. Herein, we refer to the entity of IgG4-related urinary system involvement as IgG4-related urinary disease (IgG4-RUD). In addition to the above lesion types, perirenal and periureteral fibrosis can cause complications of hydronephrosis, leading to chronic or acute kidney injury, which may require immediate medical intervention, such as double-J-tube drainage. However, recent studies have mainly focused on renal parenchymal lesions and retroperitoneal fibrosis (RPF), while little attention has been paid to renal pelvis, ureter, or bladder lesions.

Here, we present the clinical, radiological, and pathogenic features and treatment response of 65 Chinese IgG4-RUD patients from a single centre to give a brief description of the clinical spectrum of IgG4-RUD. We excluded patients with only retroperitoneal fibrosis in this study.

## 2. Materials and Methods

### 2.1. Patient Enrolment

All patient data were collected from an IgG4-RD prospective cohort study of the Peking Union Medical College Hospital in China, which was conducted from January 2011 to October 2019. All participants fulfilled both the 2019 American College of Rheumatology/European League Against Rheumatism classification criteria for IgG4-RD [18] and the 2011 comprehensive diagnostic criteria for IgG4-RD diagnostic criteria (definite, probable, and possible) [19]. All patients signed informed consent forms.

For urinary system involvement of IgG4-RD, patients needed to fulfil at least one of the following criteria: (1) histopathology: plasma-cell-rich tubulointerstitial nephritis, with >10 IgG4-positive plasma cells/HPF, and tubular basement membrane immune complex deposits; (2) imaging: small peripheral low-attenuation cortical round or wedge-shaped lesions, parenchymal masses or nodules, diffuse patchy involvement or bilateral diffuse enlargement of the kidneys, and a renal pelvis mass or a thickening of the ureteral wall or the soft tissue around the ureter; and (3) urinalysis: for IgG4-RPF patients, we enrolled only those with confirmed comorbid urine tubular lesions with urine tubular injury markers and urine protein quantification > 0.5 g per 24 hours.

We excluded patients with incomplete clinical data and the suspicion of other autoimmune disorders [e.g., Sjogren's syndrome, antineutrophil cytoplasmic antibody- (ANCA-) associated vasculitis, and sarcoidosis], suspicion of malignancy (lymphoma, kidney cancer, and metastatic carcinoma), and infection. In addition, ureteral obstructive lesions caused only by RPF were excluded. Finally, 65 IgG4-RUD patients were enrolled.

### 2.2. Clinical Data, Laboratory Tests, Imaging, and Histopathological Examinations

Demographic data, disease duration, and accompanying involved organs were collected. Routine blood examination; urinalysis and urine protein quantitation; kidney function tests; erythrocyte sedimentation rate (ESR); and high-sensitivity C reactive protein (hsCRP), complement, serum immunoglobulin (Ig), serum immunoglobulin G (IgG) subclass, total IgE, and autoantibody (rheumatoid factors, antinuclear antibodies, antiextracted nuclear antigen autoantibodies, and ANCA) levels were tested.

All patients underwent imaging examinations, such as ultrasound, CT scan, and magnetic resonance imaging (MRI). Some patients underwent positron emission tomography-computed tomography (PET-CT).

All renal needle biopsy samples were embedded in paraffin and stained with hematoxylin and eosin, periodic acid-Schiff, periodic acid-methenamine silver, and Masson's trichrome. For other biopsy samples and surgical autopsy samples, hematoxylin and eosin staining was conducted. Biopsy samples were examined by light microscopy or fluorescence microscopy. Antibodies against CD3, CD20, CD38, CD138, IgG, and IgG4 were used for immunohistological staining.

### 2.3. Assessment of Treatment Response

Clinical manifestations; laboratory tests such as urinalysis and 24-hour urine protein (24hUP) quantitation; liver and kidney function tests; ESR; and hsCRP, complement, serum Ig, serum IgG subclass, and total IgE levels were evaluated at months 1, 3, and 6 and every 6 to 12 months afterwards.

### 2.4. Statistical Analysis

Statistical analysis was performed using SPSS version 21.0 (IBM Inc., Chicago, IL). The Student *t*-test was used for the analysis of continuous, normally distributed data, while the Mann-Whitney *U* test was used for the analysis of continuous nonnormally distributed data. One-way ANOVAs were used to analyse categorical variables for normally distributed data, and the Kruskal-Wallis test was used to analyse nonnormally distributed data. A *P* value less than 0.5 was considered statistically significant.

## 3. Results

### 3.1. Demographic and Clinical Features

Among the 65 enrolled IgG4-RUD patients, 21 were diagnosed with TIN (32.3%), 5 with IgG4-related glomerular nephritis (GN, 7.7%), 3 with both TIN and glomerular nephritis (GN+TIN, 4.6%), 14 with renal pelvis or ureter involvement (21.5%), and 1 with bladder involvement (1.5%). Nine patients presented abnormal renal radiological findings, such as renal parenchymal nodule lesions (13.8%). Twelve patients had renal parenchymal lesions accompanied by retroperitoneal fibrosis (18.5%).

For the accompanying involved organs, the pancreas was the most commonly involved organ (20, 30.8%), followed by the submandibular gland and lacrimal gland (12, 18.5%, and 11, 16.9%, respectively).

IgG4-RUD patients were predominantly male (male : female 49 : 16), with a mean diagnostic age of 59.0 (52.5-67.0) years, and had multiple organ involvement. The mean disease duration was 1.5 (0.4-10.0) years. A total of 52.3% of patients had an allergy history (drug or food allergy, allergic rhinitis, asthma, etc.) The mean follow-up duration was 1.9 (0.9-3.1) years (Table 1).

### 3.2. Laboratory Features

All IgG4-RUD patients had elevated serum IgG4, 76.9% presented with hyperglobulinemia, and 92.3% had hyper-IgE.

Compared with other types, patients with IgG4-TIN presented with markedly elevated serum IgG1 [12400 (9020-15500) mg/L] and high serum IgG4 concentration [17500 (10536-30950) mg/L] and tended to have renal dysfunction with different degrees of proteinuria. The mean serum creatinine (SCr) was 151.5 (78.3-256.8) *μ*mol/L, and the mean 24-hour urine protein was 0.68 (0.35-1.41) g/24 h. No anaemia, Fanconi syndrome, or renal tubular acidosis was observed. Hypocomplementemia was another important serological feature of TIN; 94.3% of IgG4-TIN patients had low levels of C3, and 61.3% had hypo-C4. The mean C3 concentration of IgG4-TIN patients was 0.482 (0.430-0.739) g/L, which was significantly lower than that of other patients (*P* < 0.05). The mean serum C4 concentration was 0.065 (0.028-0.145) g/L.

IgG4-related glomerular nephritis patients mainly had membranous nephropathy in our study. Patients presented with oedema and nephrotic syndrome. The mean 24-hour urine protein was 11.1 (6.3-16.7) g/24 h, and the mean serum albumin was 18 (14-25) g/L. However, most patients had normal SCr [75.0 (59.0-127.5) *μ*mol/L]. Their serum IgG4 level was also markedly elevated to 10270 (8310-2950) mg/L. One patient showed low serum C3 and C4, while others had normal complement. Negative serum antiphospholipase 2 receptor (PLA2R) antibody was essential for the exclusion of primary membranous nephropathy.

Patients who presented with abnormal renal imaging seemed to be clinically quiescent with mild serology and negative urinalysis. Renal lesions were occasionally found by physical examinations. The mean SCr concentration was 60 (58-77) *μ*mol/L, with a mean serum IgG4 concentration of 9510 (4295-15050) mg/L and a mean IgG concentration of 19.9 (16.0-31.1) g/L.

Patients with IgG4-related renal pelvis or ureter involvement presented with a renal pelvis mass or a thickening of the ureteral wall or the soft tissue around the ureter, which may lead to ureteral obstruction or hydronephrosis. The mean SCr was 67.0 (63.0-78.5) *μ*mol/L at admission, and the mean IgG4 was 7920 (3058-38150) mg/L. All patients had normal complement concentrations.

Patients with renal parenchymal involvement and retroperitoneal fibrosis came to the clinic for acute renal dysfunction with a significantly elevated mean SCr concentration of 502.0 (231.5-720.0) *μ*mol/L (*P* < 0.001) as well as a remarkably elevated mean serum IgG4 concentration [14100 (3200-22000) mg/L]. The mean urine protein level was 0.99 (0.68-1.27) g/24 h. Hypocomplementemia was not a common feature in these patients (Table 2). Eight (66.7%) patients had unilateral or bilateral ureteral obstruction as well as hydronephrosis.

One patient who had bladder involvement came to the clinic for acute urinary retention, and the parotid gland and orbit were also involved. Urine protein and SCr levels were within normal ranges, but IgG4 was elevated to 36700 mg/L, IgG to 36.04 g/L, and *γ* globulin to 36.7%.

### 3.3. Imaging Features

**Figure 1:**
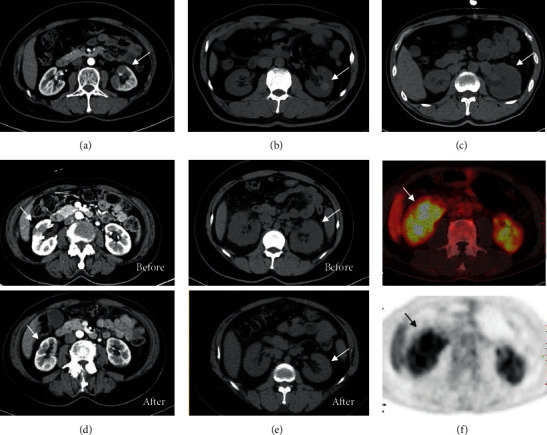
(a) Patchy low-density lesion in contrast-enhanced CT scan of IgG4-RKD (arrow). (b) Nodule lesion in renal parenchyma of IgG4-RKD (arrow). (c) Arrow is pointed at the thickening perirenal capsule of an IgG4-RKD patient. (d) Repeated contrast-enhanced CT scan revealed radiological remission of an IgG4-RKD patient after 1-year treatment. Arrow is pointed at the low-density lesion area before and after treatment. (e) Repeated CT scan of IgG4-RKD after 2 years. The swelling of the bilateral kidney prominently ameliorated. (f) PET-CT of an IgG4-RKD patient presenting with unilateral renal mass with high uptake of 19F-FDG (arrow pointed at the mass).

**Figure 2:**
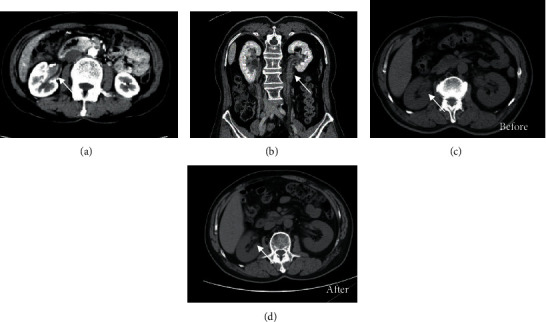
(a) CT scan revealed renal pelvis mass and thickening wall without enhancement (arrow). (b) Ureter mass without enhancement of IgG4-related ureter disease (arrow). (c) Repeated CT scan showed remission of thickened right renal pelvis wall after 3-month treatment. Arrow pointed at the pelvis lesion.

Due to renal dysfunction, only some IgG4-RKD patients received contrast-enhanced CT scans. Imaging characteristics of IgG4-RKD include a parenchymal pattern and a renal pelvis and/or ureter pattern. Three types of characteristic image features of IgG4-RKD were revealed: (1) bilateral renal cortical patchy, low-density lesions (Figure 1(a)); (2) isolated renal parenchymal mass/nodules (Figure 1(b)); and (3) bilateral diffuse enlargement of the kidneys or a thickened perirenal capsule (Figures 1(c) and 1(e)). Though it is sometimes difficult to differentiate IgG4-RD from malignancies, patients with IgG4-RD showed satisfactory radiological response to immunosuppressant treatment. For those patients with normal serum creatinine at diagnosis, renal morphology showed dramatic remission after treatment (Figures 1(d) and 1(e)). However, in most IgG4-TIN patients with chronic renal dysfunction and bilateral renal atrophy, their renal function could not return to normal even under intensive treatment. In addition to CT scans, high 18F-FDG uptake in the bilateral renal cortex also had screening and diagnostic value for IgG4-RKD, especially for patients with normal kidney function (Figure 1(f)).

Apart from renal parenchymal lesions, IgG4-related renal pelvis, ureter, and bladder lesions also presented with a solid mass or wall thickening without enhancement (Figures 2(a) and 2(b)). For those who did not undergo surgery, repeated CT scans after glucocorticoid treatment also revealed prominent improvement (Figure 2(c)).

### 3.4. Pathological Features

**Figure 3:**
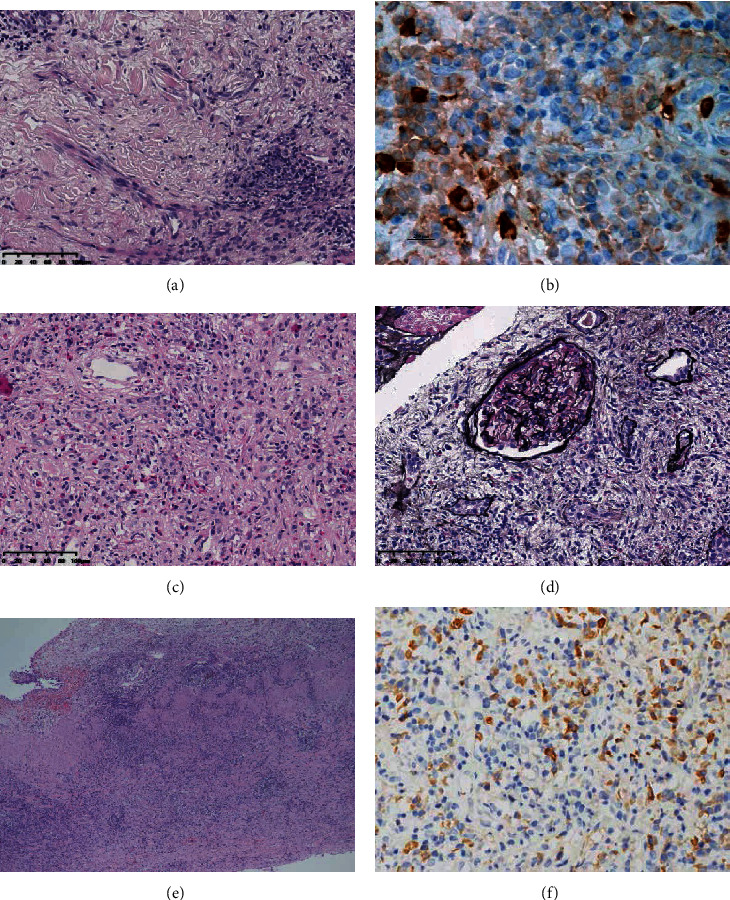
(a) Infiltration of plasma cells and lymphocytes and storiform fibrosis in renal interstitium (hematoxylin and eosin staining ×200). (b) IgG4 staining of renal biopsy (×200). (c) Eosinophil infiltration in interstitial of IgG4-TIN (hematoxylin and eosin staining ×200). (d) IgG4-MN accompanied with TIN. Storiform fibrosis can be observed (periodic acid-methenamine-silver staining ×200). (e) Diffuse infiltration of plasma lymphocytes in thickened ureter wall of IgG4-related ureter disease (hematoxylin and eosin staining ×100). (f) IgG4 staining in IgG4-related bladder disease (HE stain ×400).

Biopsies were carried out in 36 (55.4%) IgG4-RUD patients before treatment, of whom 20 (55.6%) received renal needle biopsies, 3 (8.3%) underwent renal operation, and 1 (2.8%) patient had bladder wall biopsy. Others received biopsies at other involved organs, such as the submandibular glands, lymph nodes, and intestine. Diffuse plasma cell and lymphocyte infiltration and storiform fibrosis were typical pathological features found in renal biopsy samples from IgG4-TIN patients, together with an IgG4/IgG-positive plasma cell ratio > 40% and >10 IgG4-positive plasma cell high-power field (Figures 3(a) and 3(b) ). Eosinophils were sometimes found in the interstitial area in TIN biopsy samples (Figure 3(c)). However, obliterative phlebitis was seldom observed in IgG4-RKD patients. For immunofluorescence staining, C1q, C3, or IgG deposits could be found at the tubular basement membrane area.

For renal glomerular lesions, it is difficult to differentiate IgG4-MN from primary MN merely through the analysis of renal biopsy samples, in which all had IgG4 deposition in mesangial areas, unless glomerular lesions were accompanied by typical IgG4-TIN pathological lesions or were negative for anti-PLA2R antibody under immunohistochemical staining (Figure 3(d)).

For renal pelvis, ureter, or bladder involvement, plasma-lymphocyte-rich infiltration, storiform fibrosis, and obliterative phlebitis were easily observed. An IgG4/IgG-positive plasma cell ratio > 40% and >40 IgG4-positive plasma cells/HPF were sufficient for diagnosis (Figures 3(e) and 3(f)).

### 3.5. Treatment Responses

**Figure 4:**
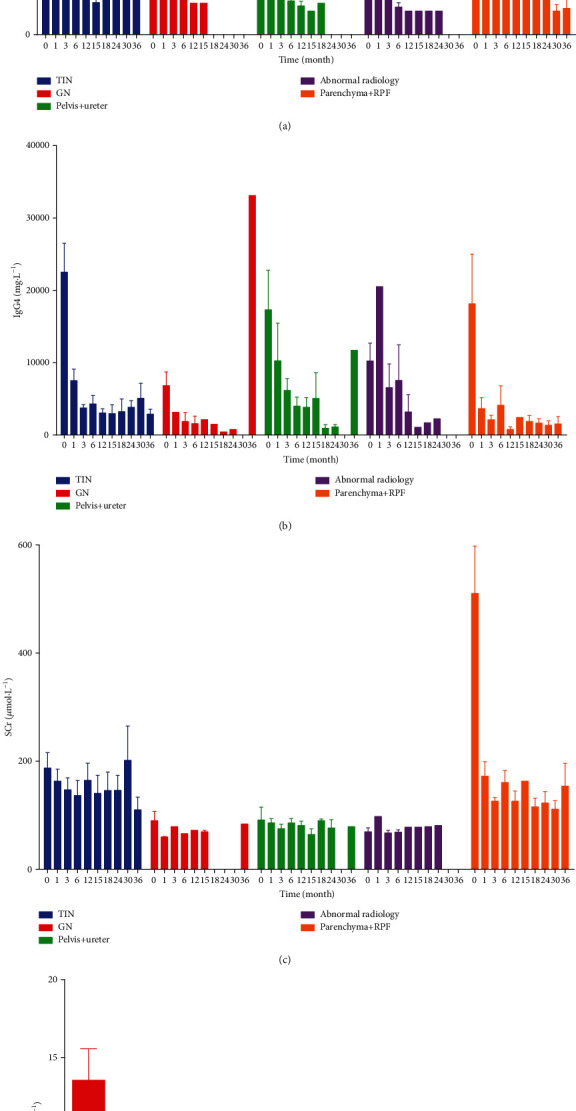
Treatment responses of IgG4-RUD.

IgG4-RUD patients showed good initial treatment response to glucocorticoids (GC). We started with 40~60 mg prednisone per day (0.6~0.8 mg/kg/d) for 1 month (except in one patient with renal pelvis involvement, who received intravenous prednisone at 200 mg per day for one week), which was then tapered to 5 mg every two weeks until 20 mg per day was reached. GC was gradually tapered 2.5~5 mg every 2 to 4 weeks until ≤10 mg/d was reached for long-term maintenance, except in one patient who was maintained at 12.5 mg/d and in one patient in whom prednisone was withdrawn 6 months after therapy (Figure 4(a)). Immunosuppressant agents, such as cyclophosphamide, mycophenolate mofetil, cyclosporine, azathioprine, and tripterygium glycoside, were combined with GC. For patients with RPF, four patients (30.8%) received double-J-catheter drainage before immunosuppressant treatment, which was removed within six months after treatment.

Serum IgG4 showed satisfactory decline after initial treatment but was reelevated at 36 months in IgG4-MN and IgG4-related renal pelvis patients (Figure 4(b)). For IgG4-TIN patients, serum creatinine declined after the first month after therapy but fluctuated later. Complete recovery of renal function could not be achieved, though serum IgG4 remained relatively stable. For patients with RPF, a rapid decline in SCr was observed in the first month after immunosuppressant treatment, but SCr did not return to normal ranges (Figure 4(c)). For IgG4-GN patients, urine protein showed a rapid decline and decreased to normal after 1 year of treatment (Figure 4(d)).

Sixteen patients (24.6%) had serum IgG4 reelevation during follow-up. Four cases (6.2%) of clinical relapse were observed in IgG4-RUD patients, and all presented with exacerbated renal impairment with serum creatinine reelevation. Two cases of clinical relapse occurred in IgG4-TIN patients: one had a relapse at 30 months after prednisone withdrawal, with baseline IgG4 concentrations of 3090 mg/dL; the other had an initial serum IgG4 concentration of 36400 mg/dL and relapsed at 18 months with prednisone at 12.5 mg per day, but immunosuppressant treatment was stopped due to skin infection. The other two patients, with TIN+GN, both relapsed at 15 months, one with prednisone at 10 mg per day and cyclosporine for maintenance and the other with prednisone at 10 mg per day and tripterygium glycosides.

## 4. Discussion

In our study, we analysed the clinical, laboratory, radiological, and histological data of 65 primary IgG4-RUD patients in our prospective IgG4-RD cohort, including renal parenchymal, renal pelvis, ureter, and bladder involvement.

Patients were predominantly middle-aged and male with multiple organ involvement. The pancreas, lacrimal glands, and salivary glands were the most common accompanying involved organs.

All IgG4-RUD patients had remarkably elevated serum IgG4, most of whom presented with hyperglobulinemia and elevated serum IgE. The clinical presentations of IgG4-RUD varied among different types. IgG4-TIN was the most common lesion type in IgG4-RUD. IgG4-TIN patients came to the clinic with renal dysfunction and a low level of urine protein. Hypocomplementemia, especially low serum C3, was a noteworthy feature in IgG4-TIN but not in other lesion types. IgG4-GN includes various pathological types, such as MN and mesangial proliferative nephritis [20]. In our study, MN played a prominent role in GN lesions. Patients presented with oedema and nephrotic syndrome and with normal SCr levels and anti-PLA2R antibody negativity. Apart from the most representative kidney lesions of nephritis or nephropathy, IgG4-related renal pelvis/ureter involvement and those with abnormal images but quiescent clinical manifestations made up approximately 35% of urinary-involved patients. Although patients with only retroperitoneal fibrosis were excluded from this study, up to 20% of patients had comorbid retroperitoneal fibrosis (RPF) and nephritis confirmed by urine tubular injury markers; this group of patients had urgent onset of acute renal dysfunction and required emergency medical intervention.

Imaging examinations were important for IgG4-RUD diagnosis. Bilateral renal enlargement, a nodule or wedge-shaped mass without enhancement, and wall thickening were characteristic radiological changes seen in IgG4-RUD patients, though they were sometimes difficult to differentiate from malignancies. High uptake of 18F-FDG in PET-CT also had diagnostic value for IgG4-RUD.

The infiltration of IgG4-positive plasma cells and lymphocytes and storiform fibrosis were common histological characteristics of IgG4-RUD. Eosinophil infiltration could also be found in lesioned tissue. For IgG4-MN patients, the coexistence of interstitial inflammatory infiltration and negative anti-PLA2R staining helped in the diagnosis. Though tissue biopsy samples could not be easily acquired in IgG4-RUD patients, tissue pathology was still confirmed as the gold standard diagnostic criterion for IgG4-RUD patients.

IgG4-RUD patients showed a satisfactory response to glucocorticoid treatment, with stable kidney function, decreased urine protein and serum IgG4, and rehabilitated radiological findings. For IgG4-TIN patients, SCr declined after the first month of treatment but remained moderately elevated afterwards. For IgG4-GN patients who presented with high baseline proteinuria, urine protein declined to normal ranges after one year of immunosuppressant therapy. IgG4-related renal pelvis/ureter-involved patients seemed to have a better prognosis than those with nephritis and nephropathy. Patients with IgG4-related renal parenchymal disease plus RPF presented with more urgent clinical conditions, but after proper immunosuppressant treatment, creatinine quickly declined after one month even without catheter drainage. While 16 patients had serum IgG4 reelevation during follow-up, only 4 cases of relapses occurred at 15 to 30 months, even without the discontinuance of immunosuppressive treatment. Serum creatinine reelevation seemed to be a specific signal for disease relapse compared with serum IgG4 elevation.

In IgG4-RUD patients, the serum IgG4 concentration was much higher than the established cut-off value: the mean initial serum IgG4 concentration was 7920-17900 mg/L. As IgG4-RUD could present only with tumescent lesions, or with individual masses but quiescent clinical manifestations, radiological examinations were noninvasive methods for disease diagnosis and monitoring.

Our study had several limitations. This was a single-centre study with a relatively small population. For IgG4-RKD patients, not all underwent renal biopsies. According to inconspicuous initial manifestations, many IgG4-RUD patients had delayed diagnoses; thus, full recovery could not be achieved.

In conclusion, primary IgG4-RUD accounts for a significant percentage of IgG4-RD, with diverse clinical presentations and a high frequency of recurrence even under intensive immunosuppressive treatment. If not diagnosed in time, renal dysfunctions are unlikely to completely reverse. Initial serum IgG4 concentration is not valuable for prognosis assessment due to different lengths of disease duration and accompanying involved organs, but close monitoring of IgG4, renal function, and radiology could help in the early recognition of urinary relapse. As the pathogenesis of IgG4-RUD remains confusing, more basic studies are needed for the precise treatment and better prognosis of IgG4-RUD patients.

## Figures and Tables

**Table 1:** Baseline features of IgG4-RUD patients.

Sex, male : female	49 : 16
Age of diagnosis, years (IQR)	59.0 (52.5-67.0)
Disease duration, years (IQR)	1.5 (0.4-10.0)
Involved organs, *n* (IQR)	3 (2-5)
Follow-up time, years (IQR)	1.9 (0.9-3.1)
Allergy history, *n* (%)	34 (52.3)
Other involved organs, *n* (%)	
Pancreas	20 (30.8)
Submandibular glands	12 (18.5)
Lacrimal glands	11 (16.9)
Cholangitis	7 (10.8)
Parotid glands	4 (6.2)
Sinus	2 (3.1)
Intestine^∗^	2 (3.1)

^∗^Both patients had multiple organ involvement, and intestinal mucosa biopsy fulfilled the IgG4-RD histopathological diagnostic criteria.

**Table 2:** Baseline laboratory features of primary IgG4-RUD patients.

	TIN (*n* = 21)	MN (*n* = 5)	Renal pelvis+ureter (*n* = 14)	Abnormal radiology (*n* = 9)	Parenchymal+retroperitoneal fibrosis (*n* = 12)	*P* value
Alb, g/L (IQR)	37.0 (27.0-42.0)	18.0 (14.0-25.0)	40.5 (36.3-47.0)	40.0 (35.5-40.8)	37.0 (29.3-42.8)	0.006
SCr, *μ*mol/L (IQR)	151.5 (78.3-256.8)	75.0 (59.0-127.5)	67.0 (63.0-78.5)	60.0 (58.0-77.0)	502.0 (231.5-720.0)	<0.001
24hUP, g/24 h (IQR)	0.68 (0.35-1.41)	11.13 (6.27-16.75)	∗	∗	0.99 (0.68-1.27)	0.002
IgG, g/L (IQR)	29.4 (19.3-41.5)	14.6 (8.9-28.7)	19.4 (11.9-37.2)	19.9 (16.0-31.1)	19.6 (17.0-32.7)	0.395
IgE, KU/L (IQR)	555.5 (341.8-913.8)	1469.0 (632.5-1943.0)	357.0 (90.7-824.3)	404.5 (100.2-649.8)	187.0 (68.6-1426.5)	0.479
IgG1, mg/L (IQR)	12400 (9020-15500)	5640 (2720-9635)	8840 (8310-12950)	8695 (6690-10993)	9110 (8730-14400)	0.015
IgG2, mg/L (IQR)	6345 (5058-8628)	3910 (3425-5045)	5440 (3840-7280)	5510 (5093-9583)	5580 (2950-7965)	0.228
IgG3, mg/L (IQR)	753 (368-1380)	154 (127-701)	678 (382-898)	525 (287-1100)	729 (434-1558)	0.193
IgG4, mg/L (IQR)	17500 (10536-30950)	10270 (8310-12950)	7920 (3058-38150)	9510 (4295-15050)	14100 (3200-22000)	0.422
ESR, mm/h (IQR)	50 (15-91)	78 (46-85)	29 (13-58)	21 (6-38)	81 (57-94)	0.046
hsCRP, mg/L (IQR)	5.92 (0.77-18.62)	2.67 (1.62-11.07)	2.14 (0.93-4.15)	1.91 (0.39-4.73)	6.07 (3.75-23.16)	0.206
C3, g/L (IQR)	0.482 (0.430-0.739)	0.579 (0.426-0.867)	0.954 (0.854-1.062)	0.781 (0.477-1.101)	0.928 (0.693-1.134)	0.033
C4, g/L (IQR)	0.065 (0.028-0.145)	0.208	0.176 (0.136-0.267)	0.205 (0.036-0.260)	0.233 (0.122-0.282)	0.051

We did not analyse IgG4-TIN+GN patients to avoid misleading data for Alb, SCr, and 24hUP. Data from the only patient with IgG4-related bladder involvement are also not listed in the chart above. ^∗^24-hour urine protein levels were not quantified due to negative routine urine tests.

## Data Availability

Data available on request.
